# Estimating Heart Rate from Inertial Sensors Embedded in Smart Eyewear: A Validation Study

**DOI:** 10.3390/s25154531

**Published:** 2025-07-22

**Authors:** Sarah Solbiati, Federica Mozzini, Jean Sahler, Paul Gil, Bruno Amir, Niccolò Antonello, Diana Trojaniello, Enrico Gianluca Caiani

**Affiliations:** 1 Department of Electronics, Information and Bioengineering, Politecnico di Milano, Piazza Leonardo da Vinci 32, 20133 Milan, MI, Italy; sarah.solbiati@polimi.it (S.S.); federica.mozzini@polimi.it (F.M.); 2 EssilorLuxottica, Smart Eyewear Lenses, Essilor International, 147 Rue de Paris, 94320 Créteil, France; sahlerj@essilor.fr (J.S.); gilpa@essilor.fr (P.G.); amirb@essilor.fr (B.A.); 3 EssilorLuxottica, Smart Eyewear Lab, Piazzale Luigi Cadorna 3, 20123 Milan, MI, Italy; niccolo.antonello@luxottica.com (N.A.); diana.trojaniello@luxottica.com (D.T.); 4 IRCCS Istituto Auxologico Italiano, Via Ludovico Ariosto 13, 20145 Milan, MI, Italy

**Keywords:** smart eyewear, wearables, physiological monitoring, heart rate, inertial measurement unit, quality index

## Abstract

**Highlights:**

**What are the main findings?**

- The “Essilor Connected Glasses”, using an IMU for heart rate estimation, showed high accuracy compared to ECG and smartwatch during controlled static activities.
- The quality index (QI) significantly impacted heart rate estimation accuracy, with higher QI values correlating with lower error rates.

**What is the implication of the main finding?**

- Smart eyewear with IMUs offers a viable, non-invasive alternative for opportunistic and unobtrusive heart rate monitoring during sedentary activities.
- QI thresholds can be used to optimize wearable HR monitoring systems for improved performance in different physiological states.

**Abstract:**

Smart glasses are promising alternatives for the continuous, unobtrusive monitoring of heart rate (HR). This study validates HR estimates obtained with the “Essilor Connected Glasses” (SmartEW) during sedentary activities. Thirty participants wore the SmartEW, equipped with an IMU sensor for HR estimation, a commercial smartwatch (Garmin Venu 3), and an ECG device (Movesense Flash). The protocol included six static tasks performed under controlled laboratory conditions. The SmartEW algorithm analyzed 22.5 s signal windows using spectral analysis to estimate HR and provide a quality index (QI). Statistical analyses assessed agreement with ECG and the impact of QI on HR accuracy. SmartEW showed high agreement with ECG, especially with QI threshold equal to 70, as a trade-off between accuracy, low error, and acceptable data coverage (80%). Correlation for QI ≥ 70 was high across all the experimental phases (r^2^ up to 0.96), and the accuracy within ±5 bpm reached 95%. QI ≥ 70 also allowed biases to decrease (e.g., from −1.83 to −0.19 bpm while standing), with narrower limits of agreement, compared to ECG. SmartEW showed promising HR accuracy across sedentary activities, yielding high correlation and strong agreement with ECG and Garmin. SmartEW appears suitable for HR monitoring in static conditions, particularly when data quality is ensured.

## 1. Introduction

The increase in the use and accessibility of wearable devices is revolutionizing lifestyle and healthcare management [1] by enabling the non-invasive and discreet monitoring of a number of physiological parameters. Indeed, wearables facilitate continuous tracking of users’ physical activity, as well as behavioral and physiological parameters, including heart rate (HR) [2,3], a key indicator of cardiovascular health and physiological stress. In recent years, wearable technology has become widespread among a diverse range of users, including athletes, patients, with an increasing interest in the general population in self-monitoring health parameters [4]. In parallel, global surveys have consistently listed wearable devices among the top consumer health and fitness trends since 2016 [5]. Accordingly, wearables are increasingly perceived as support tools to improve quality of life by enhancing health monitoring, promoting physical activity, and supporting mental well-being [6].

In this context, there is a wide use of photoplethysmographic (PPG) sensors embedded in wearable devices, capable of estimating pulse rate and pulse rate variability, though their accuracy can be influenced by motion artifacts, skin tone, and other physiological or environmental factors [7], thus requiring advanced signal processing and denoising algorithms [8].

Smart eyewear, with solutions including both sunglasses and prescription glasses, represents an emerging opportunity to be used as wearables with HR measuring capabilities, supported by recent data showing that over 50% of the population in many European countries wears prescription spectacles [9]. In the past years, several studies explored the possibility of measuring HR utilizing inertial measurement units (IMUs) embedded in head-mounted devices, exploiting the fact that at each cardiac cycle, approximately 10 g of blood flow is pushed by heart contraction towards the head through the carotid arteries, generating subtle cyclic movements of the head [10]. The accelerometers and gyroscopes embedded in the IMU enable the recording of head movements and vibrations, capturing a signal that is generated by head dynamics and, consequently, contain valuable cardio-mechanical information.

An early work explored the possibility of using the accelerometer (average sampling rate of 50 Hz), gyroscope (average sampling rate of 50 Hz), and camera (constant frame rate of 30 Hz) embedded in the Google Glass technology to measure physiological parameters, including HR, in 12 participants. A mean absolute error of 2.51 beats per minute (bpm) was achieved in sitting posture when using only the accelerometer signals, of 0.82 bpm when using the gyroscope signals, and of 1.19 bpm when combining information from all the sensors [11]. Furthermore, other studies explored different methods to extract HR information from the IMU embedded in head-worn devices, including virtual reality headsets, ranging from frequency domain methods exploiting algorithms based on the Fourier Transform to morphological analysis of the signal allowing beat-by-beat detection [12,13,14].

A recent study introduced the non-commercial “Essilor Connected Glasses” (EssilorLuxottica, Charenton-le-Pont, France), a smart eyewear prototype internally developed at Essilor, France, embedding mean HR-detection algorithms capable of achieving 96.56% accuracy within a ±5 bpm error range in laboratory settings, and resulting comparable to commercially available solutions such as wrist-worn photoplethysmography [15].

Accordingly, the aim of this study was to further validate the mean HR measurements obtained using the smart eyewear “Essilor Connected Glasses” prototype during several static activities mimicking sedentary behavior [16] in everyday life by comparing the obtained values to both a gold standard electrocardiograph and a commercial smartwatch for lifestyle monitoring based on PPG.

## 2. Materials and Methods

### 2.1. Materials

A summary of the characteristics of the devices used in this study is reported in Table 1. The non-commercial “Essilor Connected Glasses” smart-eyewear [15], hereinafter called “SmartEW”, was tested. It embeds a BHI160/BHI160B Bosch low-power smart IMU sensor, integrating a 3-axial accelerometer and gyroscope.

Data from the IMU were collected and further analyzed by proprietary algorithms described in Section 2.3, resulting in one value of HR representing an average over the preceding period of 24 s, of which 22.5 s represent the acquired data portion that is analyzed, and 1.5 s represents the time needed for the quasi-real-time algorithm to perform the computation. Together with each HR output, the SmartEW proprietary algorithm provides a quality index (QI) of the HRestimate, ranging from 0 (poor quality) to 100 (high quality). The QI is a numerical score derived from the quality of the acquired signal that reflects how reliable the HR estimate is.

The SmartEW was provided in two shapes, round and square, and in three sizes.

As a means of comparison with a lifestyle commercial solution, the Garmin Venu 3 smartwatch was utilized and worn tightly, though comfortably, above the ulnar styloid. This device embeds a photodiode to perform wrist PPG, resulting in measures of HR computed every second using the Garmin Elevate™ technology (Garmin Ltd., Olathe, KS, USA). Both signals from the SmartEW and the Garmin Venu 3 were collected using an ad hoc communication software developed by EssilorLuxottica and embedded in a RasPad tablet. The software allowed the operator to simultaneously start the recordings from the SmartEW and the Garmin Venu 3, and to store the resulting HR data on the tablet’s internal memory.

Finally, as a gold standard for HR measurement, the Movesense Flash (Movesense, Vantaa, Finland) sensor, embedding a 1-lead electrocardiogram (ECG) recorded at a sampling frequency of 512 Hz, was used. The device was worn on the chest using the Movesense chest belt, and ECG data were recorded through the Movesense Showcase (Suunto Oy, Vantaa, Finland) smartphone application.

### 2.2. Study Population and Design

Thirty healthy volunteers, of whom 15 were females and 15 males (age median [25th percentile; 75th percentile] 27 [25; 30] years, height 169.5 [165.3; 180] cm, weight 66.5 [57.5; 75] kg, body mass index 22.5 [20.1; 24.6] kg/m^2^), were recruited to participate in the experimental protocol. All the participants gave written informed consent to participate in the study. The experimental procedures described in this paper were in agreement with the principles outlined in the Helsinki Declaration of 1975, as revised in 2013; ethical committee approval of the Politecnico di Milano (Opinion n° 28/24, 21 May 2024) was obtained prior to the study.

The participants were randomly assigned to wear either round-shaped or square-shaped glasses, with an equal gender distribution in each group. Specifically, 8 females and 8 males were assigned to the round-shaped glasses group, while 7 females and 7 males were assigned to the square-shaped glasses group.

Particular attention was given to the choice of the correct glass size for each subject: the operator checked the following: (1) the eyewear was not slipping while worn; (2) the frame did not exceed face width; (3) the eyes remained in the top-center of the lenses. In addition, the subject was asked to confirm whether the frame was worn comfortably.

The experimental protocol, performed at the EssilorLuxottica Smart Eyewear Lab (Politecnico di Milano-EssilorLuxottica, Milan, Italy), consisted of a set of sequential (i.e., not randomized) static activities, carried out in a controlled lab setting under the tester's supervision, asking the subject to remain still during the overall signal acquisition:

- A total of 5 min while standing;
- A total of 5 min in the sitting posture;
- A total of 3 min of mental stress while sitting, induced by the 2-back version of the n-back test, performed using the online tool PsyToolkit [17,18];
- A total of 3 min of paced breathing at a fixed breathing rate of 6 s per breath (10 breaths per minute);
- A total of 5 min whilst sitting, with the back of the seat tilted at 45°;
- A total of 5 min lying down in the supine position.

Each static activity was followed by a 3 min resting period to minimize estimation artifacts due to possible HR transients in the measuring window.

### 2.3. HR Estimation Algorithm with the SmartEW

**Figure 1:**
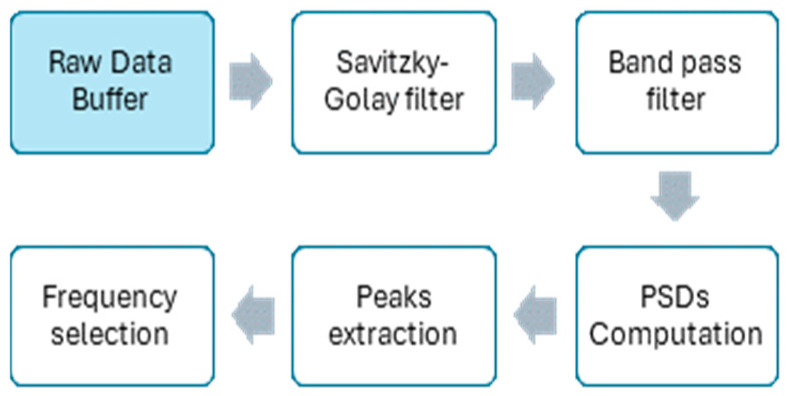
Block diagram of the algorithm for HR estimation with SmartEW.

The algorithm for the estimation of the mean HR from the SmartEW, as detailed in [15], leverages the accelerometer signals recorded from the embedded IMU, where the first step consists of buffering them for 22.5 s. Afterwards, the signals are processed on board as depicted in Figure 1, summarizing the blocks of the processing steps. First, as these signals could contain substantial noise, making direct peak detection challenging, they are down-sampled and then filtered in order to mitigate body motion artifacts. Specifically, a band-pass filter (cut-off frequencies: 0.5 Hz and 30 Hz) and a Savitzky–Golay filter [19] are applied. Additionally, a median filter is applied to remove the respiratory component. Finally, the Power Spectral Density (PSD) is used for HR estimation: the peaks of the PSD in the cardiac frequency range [0.75 Hz; 3 Hz] are assumed to be associated with HR. One of the specific difficulties of this approach is that sometimes a harmonic—which can also be in the cardiac frequency range—has a higher level of power than the fundamental one. To manage this fundamental/harmonic situation and the relevant peaks selection, a proprietary frequency selection decision algorithm was developed and applied.

### 2.4. Validation Procedure

**Figure 2:**
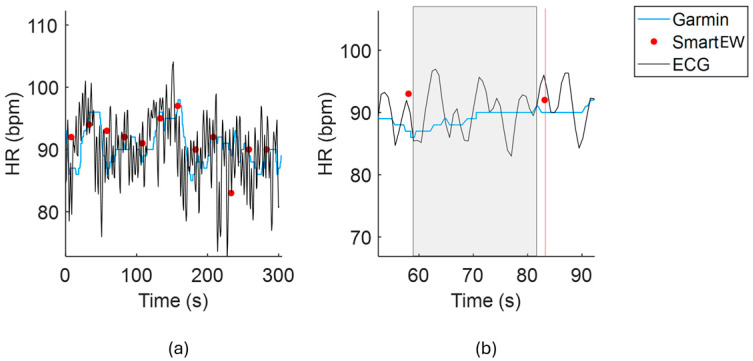
(**a**) Example of the HR derived from the Garmin (blue line), the SmartEW (red dots) and the gold standard ECG (black line) from one representative subject while sitting; (**b**) for each HR_SmartEW_ datapoint (vertical red line), the median values of HRECG and HR_Garmin_, to be used for comparison, were computed in the time window of 22.5 s width, set between 24 s and 1.5 s before each recorded HR_SmartEW_ datapoint (shaded rectangle).

The Pan–Tompkins method [20] was used to identify the R peaks on the gold standard ECG, from which beat-by-beat HR was computed. Afterwards, to allow for comparison among the HR data from the SmartEW (HR_SmartEW_), the HR from Garmin (HR_Garmin_), and the HR from the ECG (HR_ECG_), all having different HR computation windows, the values of median HR_ECG_ and HR_Garmin_ were computed over a time window of 22.5 s width, set between 24 s and 1.5 s before each recorded HR_SmartEW_ datapoint, as visualized in Figure 2.

#### 2.4.1. Comparison of HR Estimates with the Gold Standard

Statistical analysis was initially applied to compare, separately for each static activity, the HR_SmartEW_ (independently of the QI), the HR_Garmin_, and the gold standard HR_ECG_, by using the non-parametric Friedman test (*p* ≤ 0.05), with post hoc Wilcoxon signed rank test and Bonferroni correction. Non-parametric statistical tests were adopted due to the non-normal distribution of the ECG-derived HR values, as assessed using the Lilliefors test applied separately to each experimental phase (all with *p* ≤ 0.01).

The correlations of the HR_SmartEW_ and HR_Garmin_ measures with the HR_ECG_ were also assessed. Specifically, the determination coefficient (r^2^), the Spearman correlation coefficient (ρ), and the robust fit RANdom Sample Consensus (RANSAC) algorithm were computed. In particular, RANSAC represents a general parameter estimation approach applied to fit a model to experimental data that could contain outliers. By iteratively selecting random subsets of the data, inliers are discriminated from outliers and used to fit the model, thus excluding outliers from the regression and keeping the model that best represents the majority of the data [21].

A Bland–Altman analysis was then performed to evaluate the agreement between R_SmartEW_ and HR_Garmin_ [22]. Specifically, it visualizes as scatterplot the difference between paired measures against their average, thus quantifying the bias (i.e., the mean difference) between the two methods, and defining the limits of agreement (LoA) as bias ±1.96 × SD of the difference, representing a realistic error interval in which to expect a measure with one technique compared to the reference which Additionally, a paired *t*-test vs. null values (*p* ≤ 0.05) was performed to test for significance of the bias.

Finally, the accuracy of the HR_SmartEW_ estimates was calculated in accordance with the ANSI/AAMI EC13-2002 standard [23]. This standard, developed by the American National Standards Institute (ANSI) and the Association for the Advancement of Medical Instrumentation (AAMI), establishes minimum safety and performance requirements for cardiac monitors, HR meters, and alarms, which are used to acquire and/or display electrocardiographic signals used in clinical settings with the primary purposes of the continuous detection of cardiac rhythm. The standard defines a maximum tolerance of ±10% of the actual value or ±5 bpm, whichever is greater. In this study, mean HR accuracy was assessed within the ±5 bpm error range.

#### 2.4.2. Effect of the QI on HR Estimates

The relationship between the value of QI and the accuracy of HR_SmartEW_ estimates was investigated. To evaluate the impact of QI on the model error, the mean absolute error (MAE) and the Mean Relative Error (MRE) were computed as follows:

(1) $$\mathrm{MAE}=\frac{1}{n}\overset{n}{\underset{i=1}{\sum }}\left|{\mathrm{HR}}_{{\mathrm{ECG}}_{i}}-{\mathrm{HR}}_{{\mathrm{SmartHR}}_{i}}\right|$$

(2) $$\mathrm{MRE}=\frac{1}{n}\overset{n}{\underset{i=1}{\sum }}\left|\frac{{\mathrm{HR}}_{{\mathrm{ECG}}_{i}}-{\mathrm{HR}}_{{\mathrm{SmartHR}}_{i}}}{{\mathrm{HR}}_{{\mathrm{ECG}}_{i}}}\right|$$

and visualized as a function of QI.

Finally, the accuracy of the HR_SmartEW_ estimates within a ±5 bpm error range (i.e., defined as the percentage of HR_SmartEW_ values that differ by no more than ±5 bpm from the HR_ECG_), the error compared to the gold standard measurement, and the coverage (i.e., the percentage of total data that meets the QI threshold) were calculated and visualized as functions of the QI in order to determine the QI threshold that optimizes coverage while enhancing accuracy and minimizing error.

## 3. Results

The results are presented as median [25th percentile; 75th percentile], unless otherwise specified.

Data from one subject were discarded due to acquisition issues; thus, the analyses were completed on 29 subjects (15 females and 14 males).

The initial analysis included all the values of HR, thus without considering the value of QI given for each time window analyzed. The values of HR_ECG_, as computed by the gold standard, ranged from 42.2 to 128.5 bpm, thus potentially covering the full range of nominal resting HR.

### 3.1. Comparison with the Gold Standard ECG

**Figure 3:**
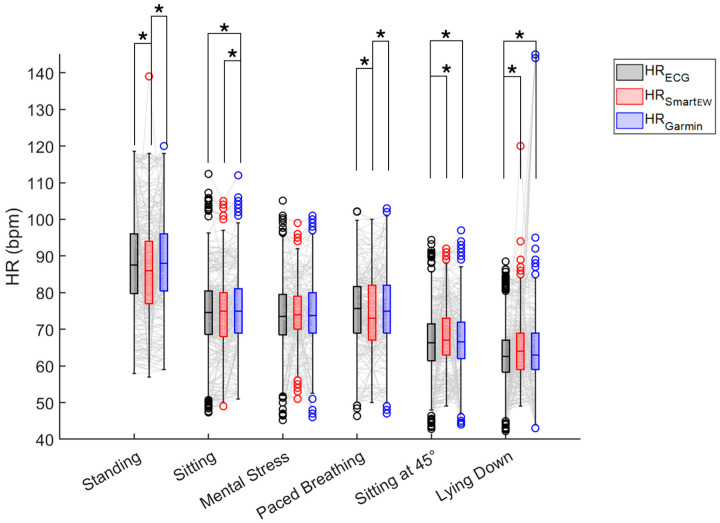
Box and whisker plot of the HR_ECG_, HR_SmartEW_, and HR_Garmin_ in the studied population for each phase of the experimental protocol. All HR_SmartEW_ values were included in this analysis, independently of the QI associated with the relevant time window. Lines connecting the values of HR relevant to the same subject and measured with the three devices are displayed in gray. Circles represent outliers. The results of the statistical analysis (Friedman test, *p* ≤ 0.05, post hoc Wilcoxon signed rank with Bonferroni correction) are reported when significant: the symbol “*” indicates a statistically significant difference (adjusted *p* ≤ 0.05) between the pair of compared methods.

Values of HR_SmartEW_, HR_Garmin_, and the gold standard HR_ECG_ were compared, separately for each experimental phase. For this analysis, all the HR_SmartEW_ values were included, independently of the associated QI values. The results of the comparison are reported in Figure 3. During sitting and mental stress, the HR_SmartEW_ values were comparable to the gold standard HR_ECG_. Differences were observed in the HR_Garmin_, both with the gold standard (−0.3 [−2.0; 1.3]%) and the HR_SmartEW_ (0.0 [−2.3; 1.3]%), while sitting. During standing and paced breathing, the HR_SmartEW_ showed an underestimation compared to the gold standard, respectively, by −0.4 [−2.1; 1.1]% and −0.7 [−3.2; 1.7]%, while no differences were observed between HR_Garmin_ and HR_ECG_.

The results of the correlation analyses are reported in Table 2. The HR_SmartEW_ provided different levels of linear correlation (r^2^) in relation to the different phases, with the best results during sitting (r^2^ = 0.92) and paced breathing (r^2^ = 0.85). Higher values of correlation were observed with the Spearman correlation coefficient, with ρ ≥ 0.70 in all the phases and up to 0.95 while sitting. Similar results were obtained with the RANSAC index, showing high correlation (up to 0.97) in all the experimental phases. The Garmin showed high correlation values in all experimental phases (r^2^ ≥ 77, except r^2^ = 0.05 in lying down, ρ ≥ 0.77, and RANSAC ≥ 0.87).

**Figure 4:**
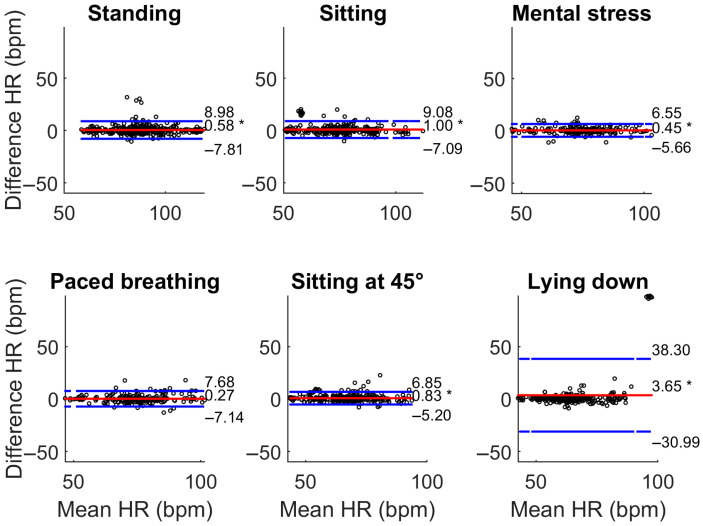
Bland–Altman analysis comparing HR_Garmin_ with HR_ECG_. Bland–Altman plots display the differences between two measurement methods against their average, allowing for assessment of systematic bias (red line) and defining the respective LoA (blue lines) as ±1.96 × SD of the difference. *: *p* < 0.05, paired *t*-test vs. null values (i.e., significant bias).

The results of the Bland–Altman analyses are reported in Table 3. In sitting and mental stress, HR_SmartEW_ resulted in non-significant biases, with comparable or only slightly lower bias and narrower LoA in the sitting phase with respect to Garmin. Significant, though acceptable, biases were observed for the SmartEW also during paced breathing (−0.97 bpm), standing (−1.83 bpm), sitting at 45° (+1.85 bpm), and lying down (+2.24 bpm). While statically seated, the LoA ranged between −7 and 7 bpm, comparable to what was obtained with the Garmin. LoAs of the SmartEW progressively increased in paced breathing (ranging −10 ÷ 8, where the symbol “÷” indicates the range), sitting at 45° (−12 ÷ 16 bpm), lying down (−14 ÷ 19 bpm), and standing (−21 ÷ 17 bpm). Garmin provided acceptable LoA in all the activities, except when lying down, attributable to outliers in mean HR, as shown in Figure 4, although resulting in a small but significant overestimation for all the experimental phases, except paced breathing. During all the phases, the SmartEW provided high values of accuracy within the ±5 bpm error margin, which were comparable to the Garmin.

**Figure 5:**
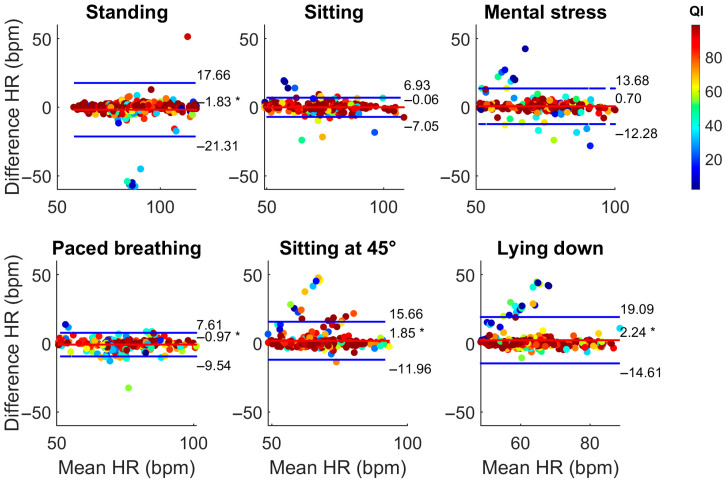
Bland–Altman analysis comparing HR_SmartEW_ with HR_ECG_. Color code indicates SmartEW QI values, from 0 (the lowest quality, in blue) to 100 (the highest quality, in red). Bland–Altman plots display the differences between two measurement methods against their average, allowing for assessment of systematic bias (red line) and defining the respective LoA (blue lines) as ±1.96 × SD of the difference. *: *p* < 0.05, paired *t*-test vs. null values (i.e., significant bias).

An additional evaluation was performed considering the QI values associated with the respective HR_SmartEW_. Interestingly, as illustrated in Figure 5, the HR_SmartEW_ values with lower QI exhibited larger differences from the gold standard, thus highlighting a clear relationship between the QI value and the accuracy of each HR_SmartEW_ measurement.

### 3.2. Selection of the Optimal QI

**Figure 6:**
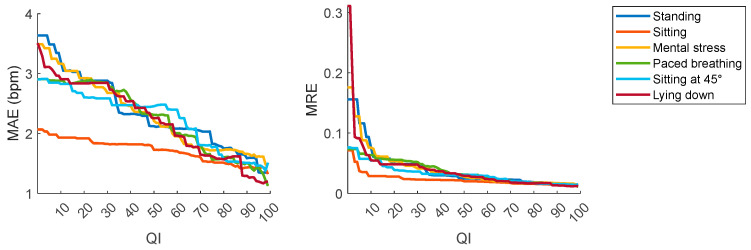
Impact of the quality index on the mean absolute error (MAE) and on the Mean Relative Error (MRE) during each experimental phase.

To further investigate the relationship between QI value and measurement accuracy, and to quantify the possible role of QI on error metrics, the MAE and the MRE were visualized as functions of QI. As shown in Figure 6, both MAE and MRE decreased across all the phases of the protocol as QI increased, with lower values registered in the sitting phase. While MRE clearly tended to plateau at low error levels for QI values of 70 and above, a trend of decreasing MAE with increasing QI was visible.

Additionally, both the accuracy within the ±5 bpm range and the difference between the HR_ECG_ and the HR_SmartEW_ (i.e., bias) were visualized as functions of the coverage (%).

**Figure 7:**
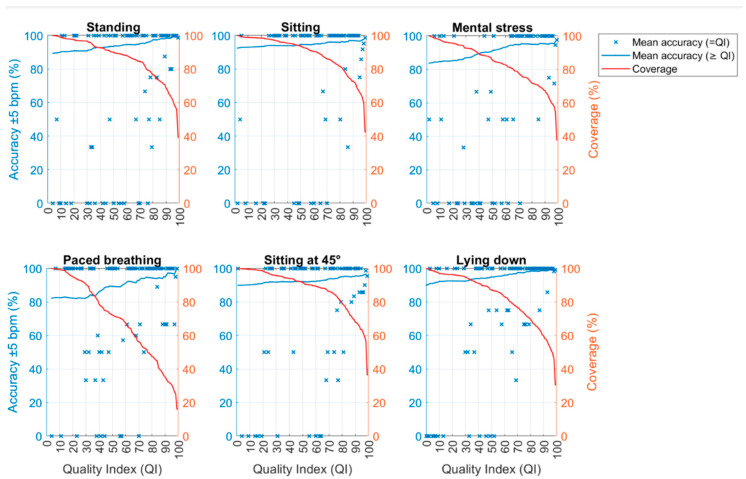
Accuracy and coverage plotted as functions of the QI for each experimental phase. Specifically, the mean pointwise accuracy at the exact QI level (i.e., “=QI”) is presented (intended as the average accuracy for the HR_SmartEW_ values associated with a specific QI), together with the mean accuracy computed for all the HR_SmartEW_ values associated with a QI equal or greater that the one on the abscissa (i.e., “≥QI”). Coverage considering QI values ≥ a given threshold is also reported.

Figure 7 shows for each phase the accuracy within the ±5 bpm and the coverage, both as a function of QI. Specifically, the mean accuracy at the exact QI level (i.e., “=QI”) is presented (intended as the average accuracy for HR_SmartEW_ values associated with a specific QI), together with the mean accuracy computed for all the HR_SmartEW_ values associated with a QI equal or greater that the one on the abscissa (i.e., “≥QI”). As expected, the mean accuracies increased with increasing QI.

**Figure 8:**
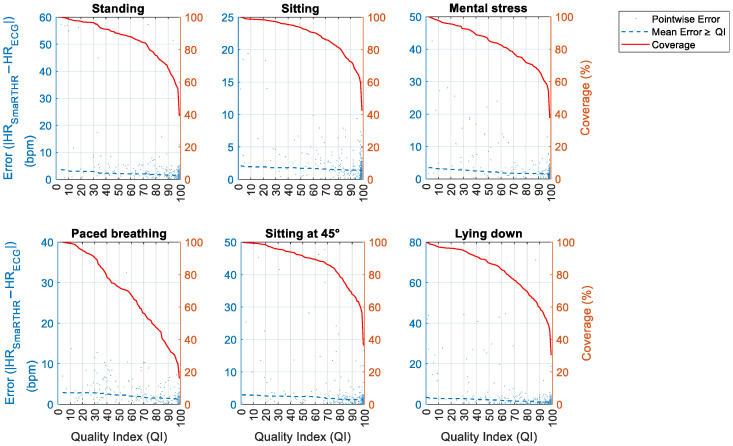
Bias and coverage as functions of the QI for each experimental phase. The bias (i.e., the absolute value of the difference between HR_SmartEW_ and HR_ECG_ in bpm) is shown both as pointwise bias (specific to exact QI values) and average bias (considering QI values ≥ a given threshold). Coverage considering QI values ≥ a given threshold is also reported.

In Figure 8, the bias and the coverage are presented as a function of the QI. In activities like sitting, standing, mental stress, and lying down, a QI threshold of 70 appeared to be beneficial to obtain low biases (within the ±5 bpm) while also maintaining a good coverage (84.36%, 86.03%, 77.72%, and 76.54%, respectively). Similarly, in paced breathing, a QI threshold of 70 allowed us to obtain low biases (within the ±5 bpm), although with a reduced coverage (55.71%). In sitting at 45°, a QI threshold of 80 allowed us to obtain pointwise errors always within the ±5 bpm, with a coverage of 78.00%, whilst a QI threshold of 70 included larger pointwise errors (up to 13.82 bpm) and a coverage of 86.55%.

**Figure 9:**
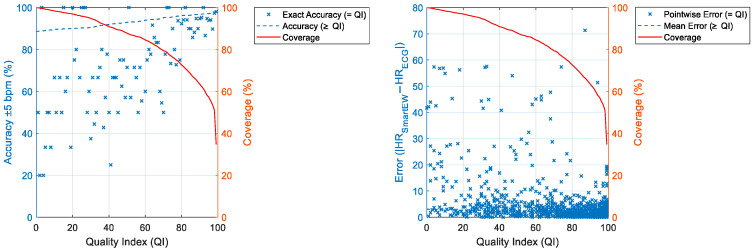
Accuracy ±5 bpm and coverage (**left**), as well as bias and coverage (**right**), are presented as functions of the QI calculated across all the phases combined.

A summary of the accuracy and the biases calculated across all the phases combined, plotted together with the relevant coverage as a function of the QI, is presented in Figure 9 and commented on in Table 4.

From these results, it emerged that a value of 70 for the QI threshold appears to be a good trade-off between reaching high accuracies with low errors while maintaining an acceptable coverage. Accordingly, the following analysis will consider only HR_SmartEW_ values with a QI ≥ 70.

### 3.3. Impact of QI on SmartEW Accuracy Performance

**Figure 10:**
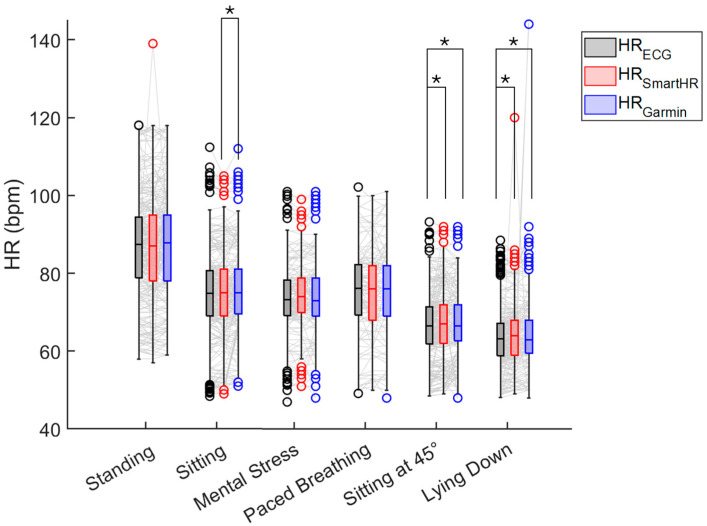
Distribution of the HR_ECG_, HR_SmartEW_, and HR_Garmin_ during each phase of the experimental protocol, considering only HR_SmartEW_ datapoints with corresponding QI ≥ 70. Lines connecting the values of HR relevant to the same subject and measured with the three devices are displayed in gray. Circles represent outliers. The results of the statistical analysis (Friedman test, *p* ≤ 0.05, post hoc Wilcoxon signed rank with Bonferroni correction) are reported: the symbol “*” indicates a statistically significant difference (adjusted *p* ≤ 0.05) between the pair of compared methods.

Figure 10 shows the distribution of HR_ECG_, HR_SmartEW_, and HR_Garmin_ when considering only HR_SmartEW_ values with QI ≥ 70. No differences compared to the gold standard were observed in standing, sitting, mental stress, and paced breathing. Finally, differences compared to the gold standard were observed in sitting at 45° and in lying down, both with the SmartEW (respectively, 0.4 [−0.9; 2.1]% and 0.4 [−1.2; 1.8]%) and with the Garmin (respectively, 0.5 [−1.0; 2.3]% and 0.3 [−1.1; 1.9]%).

All the previously computed correlation indices increased in this case, as displayed in Table 5. Similarly, as shown in Table 6, lower biases and narrower LoAs, as well as increased accuracy in all the activities, were achieved. Moreover, selecting an appropriate QI threshold led to accuracy levels in the HR_SmartEW_ values comparable to those of the Garmin device.

## 4. Discussion

In the real world, sedentary behavior, defined as any activity while awake, in a sitting or reclining posture, with energy expenditure ≤1.5 metabolic equivalents [16], represents about 60% (8.5–9.6 h) of the awake time in an adult population [24]. Sedentary behavior allows for a real-life application scenario of the opportunistic and nonintrusive monitoring of HR using smart eyewear and IMU technology, otherwise sensitive to motion artifacts, in people wearing glasses. Accordingly, this work aimed at testing the performance of a non-commercial smart eyewear in estimating the HR of the wearer while performing a set of daily life static activities in a laboratory setting.

The algorithm utilized for HR estimation provides one value of HR_SmartEW_ every 24 s, coupled to a quality index representing the reliability of each estimate. High accuracy in estimated mean HR using the SmartEW was reached, with comparable values to the gold standard ECG and to the tested commercial Garmin Venu 3 smartwatch PPG-based solution. Interestingly, in sitting at 45° and lying down, both Garmin and the SmartEW resulted in different values from the gold standard. As regards the SmartEW, this is likely due to the additional constraint introduced when the head was in contact with a surface (in this case, the chair), potentially limiting its movements in the 3D space and thus worsening the performance of the SmartEW estimation algorithm.

The conducted analyses highlighted the critical importance of setting a rejection threshold on each estimated HR_SmartEW_ considering the provided corresponding QI value, thus allowing us to discard less accurate measurements while retaining the more reliable ones. Indeed, a relationship between the HR_SmartEW_ estimation performance and the QI was observed, with higher QI values being associated with lower MAE, MRE, bias, and increased accuracy. Based on the tested SmartEW algorithm, a QI threshold equal to 70 emerged as an effective trade-off, maximizing the average accuracy within the ±5 bpm error range above 93% while minimizing the error to within the ±5 bpm, and maintaining a coverage of 80%. Higher thresholds of QI caused a decrease in the coverage, although not providing significant improvements in the performance. As a result, the analyses were repeated by considering only HR_SmartEW_ values having a QI equal to or greater than 70. This resulted in reduced biases and narrower LoA compared to the gold standard ECG, as well as increased correlation and accuracy.

**Figure 11:**
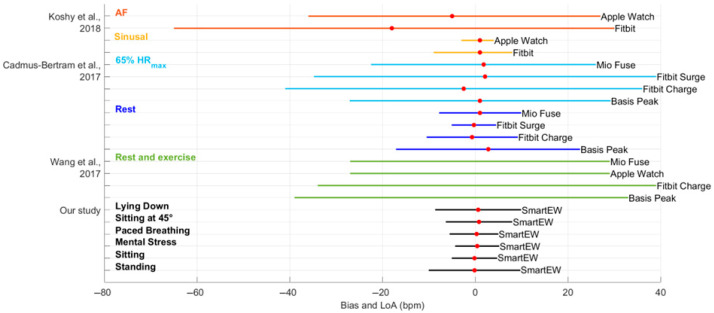
Comparison of the results obtained in this study with the state of the art [26,27,28] for smartwatches leveraging PPG technology. The results are reported in terms of bias (indicated with a red dot, when available) and limits of agreement (LoA, represented with horizontal lines) of the Bland–Altman analysis. AF: atrial fibrillation.

Current wearable consumer devices for the continuous and non-invasive monitoring of health parameters, including HR, are primarily represented by smart watches, and mainly rely on PPG technology, detecting blood volume changes through the skin by using light sensors. Several works have studied the reliability of commercial smartwatches for HR monitoring, both in resting conditions, as well as during physical exercise, and in patients with atrial fibrillation (AF), showing variable performances depending on both the technology and the subject’s state, skin pigmentation, and hairy wrist [25]. Figure 11 displays a comparison between the accuracy, expressed as bias and LoA, obtained with the SmartEW through the different phases of the experiment, after having applied the QI threshold, and that reported in the literature for several commercial smartwatches, though using different methods of analysis compared to the present work. In static activities, such as standing, sitting, lying down, mental stress, and paced breathing, the HR_SmartEW_ estimates appear comparable to the measurements obtained with smartwatches during sinus rhythm and while resting [26,27]. During exercise, the performance of smart watches was reduced, resulting in greater biases and wider LoA [26,28]. Notably, Koshy et al. [26] reported poor agreement between smartwatch and ECG-derived HR in patients with atrial fibrillation (AF), as reflected by large LoA. This is likely due to the irregular and rapid HR typical of AF, which challenges the accuracy of wrist-worn devices relying on PPG technology and the implemented software of analysis, less suited to track the highly variable beat-to-beat intervals seen in arrhythmic conditions and low perfusion states [26].

An important limitation of the current SmartEW implementation is represented by its inability to support heart rate variability (HRV) analysis, as it does not provide beat-to-beat HR data but rather average values over 22.5 s windows. This lack of temporal resolution prevents the extraction of inter-beat intervals required for standard HRV metrics [29], thus precluding its applicability in scenarios in which the goal is to study such physiological markers. Furthermore, due to the frequency-domain processing and lack of beat-to-beat resolution, the SmartEW algorithm is currently not suited for arrhythmia or AF detection, which typically requires high temporal precision and access to raw or inter-beat signals. However, we recently showed [14] that the application of different processing methods, based on template matching, could allow ECG-free beat-to-beat identification in sedentary sitting position from IMU signals embedded in smart eyewear technology. This approach, coupled with the use of rejection threshold to identify periods of sedentary activity during long-term continuous monitoring, could potentially overcome the described limitations.

Nevertheless, HR remains a clinically significant parameter, and resting heart rate in particular has been recognized as a predictor of cardiovascular outcome and overall mortality risk [30,31]. Therefore, a reliable estimation of average HR in sedentary conditions, obtained in an unobtrusive and opportunistic way, could still provide meaningful insights for health monitoring.

## 5. Conclusions

In the growing field of wearable technologies aiming at tracking health and fitness biomarkers such as HR, smart glasses emerge as a discrete and appealing solution, enabling unobtrusive and opportunistic monitoring while also offering the potential to integrate fashionable designs. The SmartEW technology analyzed herein, providing the user with average HR estimates every 24 s, resulted accurate when used in sedentary static activities, such as sitting, standing, or paced breathing, with >90% of the measurement errors within ±5 bpm compared to a gold standard ECG, and performances comparable to a commercially available smartwatch based on PPG technology. As sedentary behavior represents about 60% of the awake time in an adult population, smart eyewear technology, with IMU embedded in an already widely adopted accessory, could represent an alternative to smartwatch-based PPG measurements for the long-term monitoring of HR in the glasses-wearing population, thanks to improved wearability. However, the number of hours people wear glasses daily varies significantly based on individual needs and prescriptions, thus potentially limiting the observation window in which HR monitoring with smart eyewear could be carried out.

To go beyond the results of this study, where only static activities in lab-controlled settings under a tester's supervision have been evaluated, further improvements will need to be investigated to extend the conditions of use, including sedentary activities in real-world scenarios during a free living usage of the device, and dynamic activities. In particular, the addition of specific filtering in the preprocessing step, such as morphological filters [14], could decrease motion artifacts and allow investigating performance during dynamic activities.

## Figures and Tables

**Table 1:** Characteristics of the devices used in this study.

	SmartEW	Garmin Venu 3	Movesense Flash
Position on the body	Head	Wrist	Chest
HR sensor	Accelerometer	PPG based on Garmin Elevate™ technology	1-lead ECG
Sampling rate	200 Hz	Unknown	512 Hz
Measurement interval	~22.5 s	1 s	Beat-by-beat
Data logging	RasPad tablet	RasPad tablet	Movesense Showcase app (© Suunto Oy 2019)

**Table 2:** Results of the linear correlation (r^2^), Spearman correlation (ρ), and robust fit random sample consensus (RANSAC). All the HR values were included in this analysis, independently of the QI.

Phase	r^2^		ρ		RANSAC	
	SmartEW	Garmin	SmartEW	Garmin	SmartEW	Garmin
Standing	0.58	0.91	0.82	0.93	0.97	0.97
Sitting	0.92	0.81	0.95	0.93	0.97	0.96
Mental stress	0.67	0.93	0.85	0.94	0.93	0.96
Paced breathing	0.85	0.89	0.92	0.94	0.91	0.93
Sitting at 45°	0.54	0.90	0.80	0.94	0.97	0.96
Lying down	0.33	0.05	0.70	0.77	0.96	0.99

**Table 3:** Results of the Bland–Altman analysis (Bias [LoA]). The accuracy of the HR measurement within the ±5 bpm is also reported.

Phase	Bias (bpm)		LoA (bpm)		Accuracy (±5 bpm)	
	SmartEW	Garmin	SmartEW	Garmin	SmartEW	Garmin
Standing	−1.83 *	0.58 *	[−21.31; 17.66]	[−7.81; 8.98]	0.89	0.90
Sitting	−0.06	1.00 *	[−7.05; 6.93]	[−7.09; 9.08]	0.92	0.90
Mental stress	0.70	0.45 *	[−12.28; 13.68]	[−5.66; 6.55]	0.84	0.90
Paced breathing	−0.97 *	0.27	[−9.54; 7.61]	[−5.20; 6.85]	0.82	0.87
Sitting at 45°	1.85 *	0.83 *	[−11.96; 15.66]	[−5.20; 6.85]	0.90	0.92
Lying down	2.24 *	3.65 *	[−14.61; 19.09]	[−30.99; 38.30]	0.90	0.91

Significant bias (*p* < 0.05, paired *t*-test versus null values) is indicated as *. Specifically, the bias is defined as the difference between HR_ECG_ and HR_SmartEW_ or HR_Garmin_.

**Table 4:** Accuracy ±5 bpm, error, and coverage at QI thresholds of 50, 60, 70, 80, and 90, calculated across all the phases combined. Avg = average; Pnt = pointwise.

	QI ≥ 50	QI ≥ 60	QI ≥ 70	QI ≥ 80	QI ≥ 90
Avg. accuracy (%)	93	94	95	96	97
Pnt. accuracy (%)	>50	>50	>50 (mainly > 73)	>79	>93
Avg. error (bpm)	±2.16	±2.02	±1.75	±1.62	±1.50
Pnt. error (bpm)	Mainly < ±10	Mainly < ±10	Mainly < ±10	Mainly < ±10	Mainly < ±10
Coverage (%)	88	85	80	73	63

**Table 5:** Results of fitting HR_ECG_ and HR_SmartEW_ by linear correlation (r^2^), Spearman coefficient (ρ), and robust fit random sample consensus (RANSAC), both considering all the HR_SmartEW_ values and considering only the HR_SmartEW_ values with QI ≥ 70.

Phase	r^2^		ρ		RANSAC	
	All QI	QI ≥ 70	All QI	QI ≥ 70	All QI	QI ≥ 70
Standing	0.58	0.87 ↑	0.82	0.96 ↑	0.97	0.98 ↑
Sitting	0.92	0.96 ↑	0.95	0.96 ↑	0.97	0.98 ↑
Mental stress	0.67	0.94 ↑	0.85	0.95 ↑	0.93	0.95 ↑
Paced breathing	0.85	0.95 ↑	0.92	0.97 ↑	0.91	0.97 ↑
Sitting at 45°	0.54	0.84 ↑	0.80	0.92 ↑	0.97	0.97 ↑
Lying down	0.33	0.70 ↑	0.70	0.93 ↑	0.96	0.96 ↑

Arrows indicate whether the values increased (↑) or decreased (↓) compared to not applying QI thresholding.

**Table 6:** Results of the Bland–Altman analysis (Bias [LoA]) comparing HR_ECG_ and HR_SmartEW_ and Accuracy within the ±5 bpm error, both considering all the HR values and considering only HR values with QI ≥ 70. Additionally, the accuracy within the ±5 bpm error obtained with the Garmin is also reported. *: *p* < 0.05, paired *t*-test vs. null values (i.e., significant bias).

Phase	Bias (bpm)		LoA (bpm)		Accuracy (±5 bpm)		
	All QI	QI ≥ 70	All QI	QI ≥ 70	All QI	QI ≥ 70	Garmin
Standing	−1.83 *	−0.19 ↓	[−21.31; 17.66]	[−10.16; 9.77] ↓	0.89	0.95 ↑	0.89
Sitting	−0.06	−0.15	[−7.05; 6.93]	[−4.98; 4.68] ↓	0.92	0.96 ↑	0.90
Mental stress	0.70	0.43 * ↓	[−12.28; 13.68]	[−4.40; 5.26] ↓	0.84	0.95 ↑	0.90
Paced breathing	−0.97 *	−0.27 ↓	[−9.54; 7.61]	[−5.45; 4.91] ↓	0.82	0.93 ↑	0.87
Sitting at 45°	1.85 *	0.84 * ↓	[−11.96; 15.66]	[−6.26; 7.93] ↓	0.90	0.94 ↑	0.92
Lying down	2.24 *	0.60 * ↓	[−14.61; 19.09]	[−8.84; 10.34] ↓	0.90	0.97 ↑	0.92

Arrows indicate whether the values increased (↑) or decreased (↓), compared to not applying QI thresholding.

## Data Availability

The data presented in this study could be made available following reasonable request to the corresponding author.

## Abbreviations

The following abbreviations are used in this manuscript:

ECG	Electrocardiogram
HR	Heart Rate
HRV	Heart Rate Variability
IMU	Inertial Measurement Unit
LoA	Limits of Agreement
MAE	Mean Absolute Error
MRE	Mean Relative Error
PPG	Photoplethysmography
PSD	Power Spectral Density
QI	Quality Index
SmartEW	Smart Eyewear “Essilor Connected Glasses”
